# Next-Generation Sequencing: The Translational Medicine Approach from “Bench to Bedside to Population”

**DOI:** 10.3390/medicines3020014

**Published:** 2016-06-02

**Authors:** Mohammad Muzafar Beigh

**Affiliations:** Senior Research Fellow, National Research Centre for Plant Biotechnology, Indian Agricultural Research Institute, Pusa Road, New Delhi 110012, India; biobeigh@gmail.com; Tel.: +91-819-727-4506

**Keywords:** genomics, next generation sequencing, Whole-genome sequencing, Whole-exome sequencing, precision medicine, epigenetics, transcriptomics, bioinformatics

## Abstract

Humans have predicted the relationship between heredity and diseases for a long time. Only in the beginning of the last century, scientists begin to discover the connotations between different genes and disease phenotypes. Recent trends in next-generation sequencing (NGS) technologies have brought a great momentum in biomedical research that in turn has remarkably augmented our basic understanding of human biology and its associated diseases. State-of-the-art next generation biotechnologies have started making huge strides in our current understanding of mechanisms of various chronic illnesses like cancers, metabolic disorders, neurodegenerative anomalies, *etc*. We are experiencing a renaissance in biomedical research primarily driven by next generation biotechnologies like genomics, transcriptomics, proteomics, metabolomics, lipidomics *etc.* Although genomic discoveries are at the forefront of next generation omics technologies, however, their implementation into clinical arena had been painstakingly slow mainly because of high reaction costs and unavailability of requisite computational tools for large-scale data analysis. However rapid innovations and steadily lowering cost of sequence-based chemistries along with the development of advanced bioinformatics tools have lately prompted launching and implementation of large-scale massively parallel genome sequencing programs in different fields ranging from medical genetics, infectious biology, agriculture sciences *etc*. Recent advances in large-scale omics-technologies is bringing healthcare research beyond the traditional “bench to bedside” approach to more of a continuum that will include improvements, in public healthcare and will be primarily based on predictive, preventive, personalized, and participatory medicine approach (P4). Recent large-scale research projects in genetic and infectious disease biology have indicated that massively parallel whole-genome/whole-exome sequencing, transcriptome analysis, and other functional genomic tools can reveal large number of unique functional elements and/or markers that otherwise would be undetected by traditional sequencing methodologies. Therefore, latest trends in the biomedical research is giving birth to the new branch in medicine commonly referred to as personalized and/or precision medicine. Developments in the post-genomic era are believed to completely restructure the present clinical pattern of disease prevention and treatment as well as methods of diagnosis and prognosis. The next important step in the direction of the precision/personalized medicine approach should be its early adoption in clinics for future medical interventions. Consequently, in coming year’s next generation biotechnologies will reorient medical practice more towards disease prediction and prevention approaches rather than curing them at later stages of their development and progression, even at wider population level(s) for general public healthcare system.

## 1. Introduction

With the revolution in the socioeconomic milieu of the modern era, there has been a dramatic change in occupation, lifestyle, and nutrition of the world population affecting global health and wellbeing. The alarming rise of cancers and other metabolism-associated pathologies such as hypertension, diabetes, cardiovascular ailments, *etc.*, reflects a disparity between our diet, lifestyle, and thrifty genetic background [1]. Most of these diseases are among a group of devastating common health problems afflicting populations worldwide. These diseases are displaying strong genetic origins or links [2], and environmental exposure has been found to play a vital role in disease development and progression. Today scientists are able to sequence the entire genome at a single-nucleotide resolution level, thanks to the cheaper, faster, and increasingly accurate whole exome and whole-genome sequencing technologies [3]. A new era has started where pathologies will be defined at the molecular and/or cellular levels rather than the anatomic level; where treatments are getting personalized and more target based, rather than uniformly applied across wider population(s) [4]. Genomics, and its related technologies, have increased their reach in medical practice in recent times and its impact has become more relevant and its use conspicuous particularly in the field of oncology, infectious biology, molecular diagnosis, *etc*.

Recent genomic applications are ushering large-scale genotyping studies to detect genetic variations, comprehensive gene expression analysis to identify key molecular pathways [5], and other epigenetic signatures that have been found to be involved in various disease etiologies. Although scientists and healthcare policy-makers are still struggling to cope with the interpretation and analysis of large scale genomic data that is coming out of next-generation genome sequencing projects. Nonetheless, genome-based applications have started making rapid forays into modern clinical arena through pharmacogenomics, newborn screening, diagnostics, genetic/epigenetic testing, *etc.* [6,7]. It has been proposed that emerging trends in precision/personalized medicine will incredibly boost our abilities to explore the role of various genetic factors, as well as the effect of environmental effect on diseases (epigenetics). Genomicists are busy in discovering new biological markers for determining disease subtypes, predicting risk factors, and responses to drug treatment besides understanding the life-course of various pathologies both at the genetic and mechanistic levels [8].

With genomic discoveries being published literally on a weekly basis, collective efforts should be made to translate most of the novel genomic findings into medical interventions. Hence, there should be a persuasive need to educate medical students, physicians and healthcare policy-makers about the emerging trends in genome-based methodologies. Whole-genome and whole-exome sequencing have started getting used in the diagnosis of various cancers, intellectual disabilities, developmental delays, and autosomal/*x*-linked recessive conditions in young children [9,10], and recent genomic studies have also implicated defects in mitochondrial energy metabolism at the initial stages of diabetes [11]. There are many proven medical tests that are already using next-generation sequencing tools with high accuracy and at much lower cost, and many additional tools are also in the pipeline that are offering greater promise for future medical interventions. Currently, every baby born in the United States is tested for between 29 and 50 treatable genetic ailments through a public health program called newborn screening [12].These recent trends along with the constant increase in the number of loci contributing to genetic diseases will encourage routine use of -omics tools in future medical practice. The CRISPR (Clustered regularly interspaced short palindromic repeats) based gene-editing technique that requires highly-accurate DNA sequencing in order to replace the altered or defective variant nucleotide with the wild-type. NGS (Next Generation Sequencing) is going to prove a pivot for CRISPR-Cas 9 system since NGS methods are known to provide highly-accurate DNA sequence data and, hence, will pave the way for gene therapies for many genetic diseases.

## 2. Cancer Genome Profiling and the 40 Year War

Cancer is the leading cause of morbidity and mortality worldwide with approximately 15 million cases and 8 million cancer-related deaths reported per year [13]. Cancer is known to be caused by defects in the genome and progresses with the accretion of myriad types of mutations, including somatic mutations, somatic copy number alterations (SCNAs), and other structural variants (SVs), often accompanied by genetic and epigenetic modifications. SCNAs are considered as one of the main epithet of cancer genomes [14]. An epic US effort to genetically profile 10,000 tumors have officially recently come to an end [15] and the project which was started in 2006 as a US $100-million pilot, The Cancer Genome Atlas (TCGA) is now the biggest constituent of the International Cancer Genome Consortium that have discovered nearly 10 million cancer-related mutations [16]. The International Cancer Genome Consortium (ICGC) is another major constituent of oncogenomics research projects launched for generating the database of cancer genomes [17]. The TCGA and ICGA are making available an inclusive mutational dataset from coding regions in >12,000 cancers and the COSMIC (Catalogue of Somatic Mutations in Cancer) database is extensively curating mutations from targeted sequences [14,18]. The application of sophisticated high-throughput capabilities combined with robust bioinformatics tools has contributed to highlight the similarities and differences in the molecular edifice of each cancer and across multiple types. According to Sheila Reynolds of Institute of Systems Biology at Seattle, “The notion is to create ‘cancer genome clouds’ where researchers can share data and swiftly run *in silico* experiments as easily as a Worldwide Web search” [19]. The TCGA and ICGC will provide scientists with an enormous amount of publicly-available data about genetic/epigenetic mechanisms, molecular biomarkers, and druggable targets [20] for cancer genomes.

**Figure 1:**
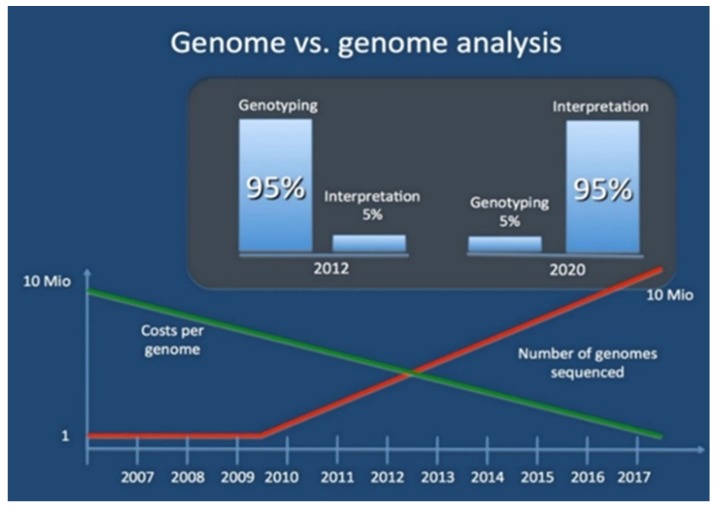
The genome and genome analysis reveal the decreasing cost of sequencing genomes and exponential growth of sequenced genomes, as well as the share of genomic data analysis in next few years [32].

The genomic era of cancer biology is evolving rapidly and is presently being driven by the advent of whole genome and/or whole exome (WE) sequencing methods, and are able to provide scientist with exquisitely sensitive and high-resolution data [21]. Both types of sequencings have been augmented by cDNA sequencing technique referred to as RNA-seq, to widely explore alterations not only at the transcriptomic, RNA-seq or gene expression levels but have also helped scientists to understand, chimeric gene fusion transcripts, aberrant splicing processes which are characteristic of most cancer cells and somatic cell mutations [22]. Single molecule or third-generation sequencing techniques addresses the shortcomings presently afflicting the current NGS platforms, e.g., single molecule real-time sequencing (SMRT) and Nanopore sequencing are becoming popular and joining the NGS platforms and are predicted to revolutionize future genomic research [23]. Hence, cancer genome sequencing got revamped from individual gene re-sequencing techniques which employed traditional PCR and Sanger sequencing methods to either targeted, whole genome, or whole transcriptome sequencing approaches. Massively-parallel genome sequencing platforms, coupled with necessary bioinformatics applications (Figure 1) are helping genomicists to generate, analyze, and interpret large-scale genomic data [17,21]. Sequencing the genomes or exomes (a protein-coding portion of the genome) of individual patients or populations, is becoming a collective approach for finding the causative genetic variants especially in complex diseases like cancers [24]. Characterizing the genetic profile of each tumor type will be helpful in devising the effective line of treatment for each type of tumor. Luo *et al.* have published one such methodology, involving the use of collective short hairpin RNA (shRNA) screening models of cancer cell lines that identified genes essential for growth and other related phenotypes in different cancer cell lines, as well as the genes involved in response to tumoricidal agents [21]. Latest approaches like pharmacogenomics will specifically help clinicians to determine optimal dosing and/or predict toxicity for various drugs e.g., warfarin, tricyclic antidepressants *etc.* [25]. Nearly 50 FDA (Food and Drug Administration) approved drugs have labels containing information about pharmacogenomics details [19]. Next-generation genomic technologies have been able to generate ample amount of data to illustrate gene expression profiles, DNA methylation/acetylation levels, histone modifications, analysis of transcription binding factors, and other DNA regulatory protein binding sites in various disease states [26]. Bioinformaticians have also lately been able to build the datasets that can systematically characterize the wide array of genomic variations among different sets of tumor models [21]. Although the use of whole-genome sequence (WGS) is far from routine today, particularly due to problems associated with data analysis, interpretation, and storage, some of the outcomes delivered so far are lending a broader vision into the potential role of both WGS/WES approaches for future disease diagnosis and prognosis [22]. NGS platforms have the formidable ability to screen wide variety of genomic aberrations, such as single nucleotide variants (SNVs), copy number variations (CNVs), multiple nucleotide variants (MNVs), and small and large insertions and deletions of various genes [27]. Currently discoveries in clinical diagnostics outpace the development of targeted therapies [28] and is supposed to expand greatly as more targets become known. The introduction of “benchtop” NGS platforms and progressive simplification of NGS methodologies are bringing NGS-based clinical tests to routine practice, e.g., WES was used in the diagnosis of Barterr syndrome in one patient which led to a reevaluation of his treatment [29], and therapeutic decision-making for somatic cancers have been taken after revaluating the NGS test results [30]. Similarly, WES (Whole Exome Sequencing) has been found to be instrumental in identifying single nucleotide variants in circulating tumor cells especially in prostate cancer patients, hence, helping to devise therapies, as well as monitoring future relapses of cancer. The WES and WGS sequencing should be considered in case of more complex, rare undiagnosed and less informative pathological conditions [29] and, hence, expanding the scope of “personalized medicine” in future medical settings. The integrated approach will be to interpret mutations and identify driver genes by combining high-throughput cellular assays with WES and/or WGS to develop comprehensive cancer genome databases. Another popular sequencing method is that of amplicon sequencing, a technique in which selective parts of the genome are amplified/enriched and are sequenced. This method is providing a good substitute for massive WGS and is known to require a much lower amount of DNA for sequencing reactions, besides generating far more information and less data than both WES/WGS, and that, too, at a fraction of cost [31].

## 3. Upcoming Age of Personal Genome Sequencing

More than a decade after the completion of the human genome project, many in the scientific fraternity might be forgiven for thinking that genome-based medical tests would be at the forefront in medical diagnosis and treatment. However, rapid progress in next-generation sequencing technologies has started to bring genomic medicine out of dormancy and, as a result, genomic methodologies have started getting inculcated into advanced medical specialties at a rapid pace. DNA sequencing technologies are making large-scale personal genome sequencing (PGS) a rapidly-approaching reality [33] and, thus, giving rise to the field of personalized genomic medicine. NHGRI (National Human Genome Research Institute) defines genomic medicine as “an emerging medical discipline that involves using genomic information about an individual as part of their clinical care (e.g., for diagnostic or therapeutic decision-making) and the health outcomes and policy implications of that clinical use” [12]. Personalized genomic medicine (PGM) can simply be defined as sequencing of an entire genome of an individual patient and tailoring treatments based on his/her genetic make-up [34]. Personalized genome sequencing (PGS) is opening up new possibilities for patients through the individualized preventive healthcare (iPH) system. Complete human genome sequencing is now becoming available at an increasing scale and decreasing cost, thanks to fast-developing massively-parallel genome processing based on micro- and nanoarrays [33]. The current ‘Holy Grail’ in genomics is the “$1000 Genome”, which is an attempt to make sequencing and mapping of individual genome(s) as cheap as $1000 so that every individual will have a record of their genome for predicting and preventing the onset of diseases, as well as devising preventive strategies for diseases [35]. The dramatic price decrease, along with the upsurge in its applications, especially in the molecular diagnostic field, is going to herald a new phase in personal genome sequence (PGS). The data generated by NGS and other omics tools are enabling, and will further enable, clinicians to make improved diagnostic and treatment decisions in future clinics, e.g., discovery of *BRCA1*, *BRCA2* variants and other biomarkers for breast and other chronic illness have been inducted as part of the routine clinical tests for breast cancer patients, profiling genetic markers like *HER2*, *ER*, and *PR*, as well as development of molecularly targeted therapies such as trastuzumab which specifically targeted *HER2+* breast cancer patients. The identification of variants in genes like *BRCA1*, *BRCA2*, and *TP53* using the Illumina HiSeq platform is higher sensitivity than traditional diagnostic methods, hence demonstrating the effectiveness of NGS tools in cancer diagnostics and also deciphering the sophisticated mechanisms of gene-gene interactions [23]. The advent of novel sequencing technologies such as Nanopore technology is emerging as a frontline method for parallel genome sequencing methods and is contributing in establishing NGS as a relevant tool for clinical sequencing technology for future.

**Figure 2:**
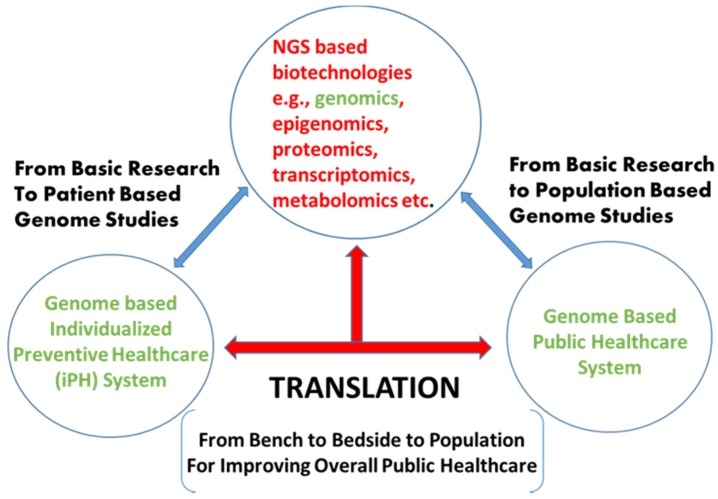
The schematic representation of next generation biotechnologies especially genome-based discoveries and their applications for patient and population-based studies for improving individualized, as well as the public, healthcare system.

One of the main goals of new age genomicists will be to usher an era of precision and/or personalized medicine by comparing normal genome biology and its perturbations during different medical conditions [36]. By removing most of the throughput and resource limitations seen with customary methods, next generation genomic applications have provided scientists with the required ability to analyze the large array of genes or genomes in a single run. Although large-scale genome sequencing is an exhilarating scheme, the real challenge lies in converting the resulting data into an actionable clinical outcomes (Figure 2). As personalized drug treatment and genomic medicine gets closer to reality, WG/WE sequencing projects should be carried out on all ethnic populations and should also be able to cover nearly all possible genetic alterations/changes present in wider populations. Genome sequencing has already started making its presence felt in the global healthcare area [37] and is rapidly making inroads into the field of molecular diagnostics. Since 2010 there has been a dramatic increase in clinical trials involving NGS tools ranging from WGS, WES to RNA-seq and targeted sequencing to find genomic variants that act as drivers for various diseases. The launching of ClinVar database at NCBI is a right step in studying, sharing the clinically-relevant variants among research groups involved in research and development of treatment for genetic diseases (GDs).

Obtaining complete genetic and epigenetic information coupled with routine transcriptome profiling and numerous other functional genomic tests will inevitably lead to the comprehensive understanding of the molecular edifice of various chronic diseases [33]. To analyze and interpret the increasing amount of sequencing data a number of statistical methods and bioinformatics pipelines have to be developed for read alignment, variant detection (point mutation, copy number variation, *etc*.), and variant functional prediction [17]. Due to the multifactorial nature of some diseases, pinpointing the cause or causes will be a daunting task [24]. Personalized genome sequencing will facilitate the identification of diverse molecular signatures both at genetic and epigenetic levels and the role environment plays in disease development and progression [12]. In the last ten years, the advancements in next-generation sequencing (NGS) technologies and target enrichment methods have resulted in the identification of genes responsible for more than 40 rare disorders [38]. Although there are certain risks associated with the PGS, the most serious being an over-interpretation of results based on limited understanding of the contextual information [33]. Therefore, utmost care needs to be taken to devise clinical strategies before employing genome-based applications for disease treatment.

## 4. Epigenetic Biology and Its Implications on Human Health

The environment has been known to play a significant role in disease development and progression, and nutrition has been particularly found to modulate gene activities at different levels with either activation or repression of key regulatory factors [39]. One of the promises of the human genome project was that it could revolutionize the understanding of the underlying causes of most of the genetic diseases by delineating the sequential arrangement of base pairs of DNA molecules [40]. It has been found that only 10% of the human diseases have genetic causes and the remaining are caused by exposure of genomes to environmental factors during the lifetime of an individual [41]. Therefore, delineating the environmental factors for disease predisposition have become a prerogative for contemporary research and NGS applications are playing a pivotal role in solving this intricate relationship. In some diseases, environmental factors can alter chromatin structure not by making changes in DNA sequences but rather by modification of chromatin or DNA, and these changes have commonly been termed as epigenetic modifications [42]. The occurrence of epigenetic markers (modifications) and other molecular signatures on the chromatin have long been identified to influence the gene expression and other associated genome regulatory mechanisms [43]. These epigenetic modifications are structural adjustments in the DNA molecules resulting from post-translational modifications (PTMs) of histone proteins such as acetylation, methylation, phosphorylation, alterations of DNA methylation levels, *etc*. These mechanisms are known to regulate transcription, response to DNA damage, histone variant exchange, as well as utilization of non-coding RNAs (lncRNAs/miRNA), *etc*. [43] in the chromatin structure. Best known epigenetic modification has been DNA methylation, as it is known to widely regulate gene expression in various cellular systems and have commonly been targeted by scientists for understanding many chronic illnesses like cancers. Epigenetic modifications has been found to be a major contributor to the germline and somatic cell mutations and has been proven by many studies. Many biochemical assays or methodologies have been used for screening DNA methylation sites in order to identify epigenetic “hotspots” [44]. Early-life stress have been identified as one of the important factor for causing epigenetic markings (e.g., DNA hyper- or hypomethylation) at important gene regulatory regions of the genome e.g., persistent increase in arginine vasopressin (AVP) expression in hypothalamic neurons have been found to be caused by DNA hypomethylation triggering alterations in behavior and neuroendocrine secretions [40]. Several drugs targeting epigenetic sites are also undergoing clinical trials to assess the post-epigenetic drug treatment effects in order to assess the effect of epigenetic targeting molecules. The search for novel biomarkers and/or epigenetic sites have been bolstered by recent advances in NGS technologies [45]. The discovery of biomarkers, especially epigenetic regulators, have become a matter of urgency as the latest discoveries are going to boost our current understanding of disease progression, drug risk assessment, and varied responses to drug treatments in different patients [45]. Nowadays, genomic tools are also enabling scientists to identify diet-induced changes at molecular genetic level. This new branch of genomics involving the study of the relationship between nutrition and genomics has been termed as nutrigenomics [19]. Today, we know that some of the non-infectious diseases like asthma, allergies, cancer, obesity, *etc*., can not only be triggered by genetic causes but also by exposure to environmental factors, even if exposed as early as embryonic development or during infancy [46]. Therefore, the possibility that these exposures can induce the onset of transgenerationally-transmitted diseases which, in turn, can have the profound effect on human health. Transgenerational epigenetic inheritance has gained increased attention due to the possibility that exposure to environmental contaminants induces diseases that propagate through generations by inducing epigenetic alterations even during gamete formation [46]. These transgenerational epigenetic changes increase the abnormal reproductive or metabolic phenotypes causing the incidence of obesity, polycystic ovary syndrome (PCOS), germ cell apoptosis, *etc*. [47]. Our current understanding of the epigenome has trailed well behind our knowledge of the genomes, since it has been very difficult to study [48]. The application of next-generation sequencing platforms from RNA libraries (RNA-Seq), chromatin immunopreciptate (CHiP-Seq), bisulfite-treated DNA sequencing, and other omic-based technologies are making it feasible to study epigenomes in a high-throughput fashion [49]. The launching of the Human Epigenome Project (HEP) by the international consortium is a positive step in that direction in order to comprehend the effect of epigenetic modifications on normal and altered human genomes [48]. Therefore, time will not be far when individual genome/epigenome analysis will become a part of overall disease assessment and treatment strategies and a new genesis of personalized genomic and epigenomic sequencing results will change the entire gamut of disease therapy in future clinics.

## 5. The ENCODE Project: Debunking the Myth of ‘Junk’ DNA in the Genome(s)

In 2003 ENCODE (ENCyclopedia Of DNA Elements) project was started by National Human Genome Research Institute (NHGRI) to identify all the functional elements present in the human genome and results were made public in 2012 with around 30 publications appearing in different Journals. ENCODE was started as a pilot project to decipher 1% of the functional elements of the human genome and has now been extended and upgraded to cover whole-genome assays in human and mouse models. To date around 5000 experimental results have been released for use by the scientific community [48]. Encyclopedia of DNA Elements Consortium (ENCODE) collaborators has identified putative functional elements including novel genes, DNA-encoded regulatory elements, RNA transcripts and other regulatory factors acting at RNA levels using the range of genomic methods based on biochemical experimental set-ups [50]. Of particular interest to researchers are projects which are mapping exposed and accessible parts of chromatin, long-distance chromatin interactions, DNA methylation sites, and other important chromatin proteins. Systematic mapping of transcription factors and its binding sites, other DNA-protein interaction sites, histone markers, 3D chromatin structure, DNA regulatory-factor occupancy patterns as well as other epigenetic signatures are providing scientists with the wealth of information about various functional aspects of genomes [51]. The data generated by ENCODE has enabled scientist to assign biochemical functions for nearly 80% of the genome, particularly to the regions lying outside the coding regions and also provided statistically important linkage between causative variants and different disease types. ENCODE projects are also proposed to significantly augment the scope of precision and/or personalized medicine and its implementation in modern clinical settings. The ENCODE program still continues to create the comprehensive catalog of functional gene elements present both in human and mouse genomes by identifying histone modifications sites, transcriptions factors, RNA-binding proteins as well as measuring the levels of DNA methylation and hypersensitivity sites [48]. The integration of overall ENCODE data will help scientists to explore the role of various candidate functional elements in disease etiologies as well as in understanding their functioning in biological systems. One of the important aspects undertaken by ENCODE project collaborators is the integration of functional elements of ENCODE with the disease-associated SNPs earlier identified by GWAS (Genome Wide Association Studies) and expression quantitative trait loci (eQTL) experiments. Since disease-associated SNPs are known to significantly affect transcription factor (TF) binding, ChIP-seq experiments performed under ENCODE program have helped us to identify novel SNPs in an allele-specific TF binding manner in any cell types and same data was compared with HapMap project and 1000 Genomes Project [52]. Based on the successes in the ENCODE projects, NHGRI has recently earmarked around $38 million for further expansion of ENCODE projects in terms of functional genomic data and integrative development of computational tools for efficient analysis, interpretation as well as utilization by research and medical fraternities [19].

High-throughput genomic technologies such as latest microarrays, next-generation sequencing (NGS), epigenomics studies and ENCODE results are introducing new paradigms in genomic medicine as well as deciphering the effect of environmental factors on disease development and progression through a new emerging field in omics technology known as Exposomics (study of the environmental exposure on genomes) [53,54].

## 6. Precision Medicine and Public Healthcare

With the euphoria surrounding the accomplishment of the Human Genome Project (HGP) in 2003, genome-based discoveries could not get implemented into clinical applications for disease treatment. However, in recent times, genomic findings have begun to get introduced into medical facilities, especially in the areas of oncology, infection biology, and other rare and undiagnosed diseases and credit goes to various NGS-based platforms. More recently people have started discussing adoption of “precision medicine” approaches in clinics where genomics, epigenomics, transcriptomics, exposomics, *etc.*, -like methodologies are helpful in disease examination, treatment, and management [12]. The development of “pharmacogenomics assistant” type of tools will assist clinicians in devising personalized drug treatment/s based on their genotype-to-phenotype pharmacogenomics data [55]. The significance of precision medicine in clinics can be gauged from President Barack Obama’s state of the union address speech in January 2015, where he stressed the importance of the precision medicine approach for solving future healthcare problems [56].

Precision medicine is an approach to medical diagnosis, treatment, and risk assessment based on an individual’s genetic makeup, gene expression patterns, *etc*. [57]. The recent data surge has started coming out of various laboratories as numerous labs have begun adopting new, and faster, equipment for sequencing DNA, including WGS/WES, RNA transcriptomic sequencing, *etc*. Sequencing of a single person’s genome is known to generate 100 gigabytes of raw data, whilst the refined and analyzed genome of one person may generate <1 GB of data. Future DNA/RNA sequencing projects of wider populations will generate terabytes of data in years to come (Figure 1). This explosion of genomic data will be helpful in implementing precision and/or personalized medicine approaches in future clinical settings. The triumph of genome sequencing has also brought many technical challenges, like analytical and interpretative calculations, ranging from validations of a large number of genetic changes in patients, and their feasibility, to managing and analyzing the terabytes of data, will be challenging tasks for the scientific community [58]. The bottlenecks in omics approaches, especially large-scale genomics, is data management, integration, analysis, as well as interpretation by genomicists and adaptation by clinicians [58].

Genome-Wide Association Studies (GWAS) have previously created fine and detailed genotypic information at high-resolution level [59] hand helping in identifying common genetic determinants in diseases [3] and that, together with omics-based applications, will create a new paradigms in contemporary medicine. As genomics is propelled by rapid advances in technology and computational proficiencies, genomic procedures are going to become a part of every medical specialty and will primarily involve applications to detect genetic variations that are associated with high-risk disease factors, as well as abnormal responses to drug treatments [60]. The scale and proficiency of sequencing that can now be achieved have reached unparalleled levels and are helping varied research areas ranging from medicine, [61] agriculture, forensic sciences, *etc*. Next generation sequencing technologies have tremendous capacity to analyze multiple genes at a time and have begun to replace Sanger sequencing, pyrosequencing, and real-time PCR-like methodologies are widely being employed in various laboratories [62]. The interaction of multiple factors including genes, non-coding RNAs, as well as proteins in cellular systems, are known to form complex networks of biomolecules known as interactome which, along with a systems biology approach, will widely be explored while devising distinctive contours for future therapeutic interventions. Future doctors and health care providers will rely more on a kind of internet-of-DNA in which medical professionals will search for better drugs and effective lines of treatment through dedicated online web networks like Amazon, Google, *etc.* [63].

## 7. Conclusions and Future Challenges

The modern medicine has made tremendous contributions to the public healthcare and well-being, e.g., from the discoveries of antibiotics to vaccine developments, in addition to important medical discoveries and surgical interventions. However, to what extent it has helped to reduce morbidity and mortality patterns, as well as what impact it has made to wellbeing and life expectancy, is still a debatable question [43]. Scientists have now begun to understand the interconnections between genetics and diseases more accurately and extensively than before. Technological advancements like NGS has made rapid inroads in our understanding of the genetics, environment and occupational diseases [64]. The success of genomic and ENCODE projects has added new dimensions to the implementation of precision and/or the personalized medicine approach and is hopefully going to herald a new era in public healthcare. Therefore, the term “Precision Public Health” will be an appropriate phrase to complement the developments and advancements in NGS applications for public healthcare.

**Figure 3:**
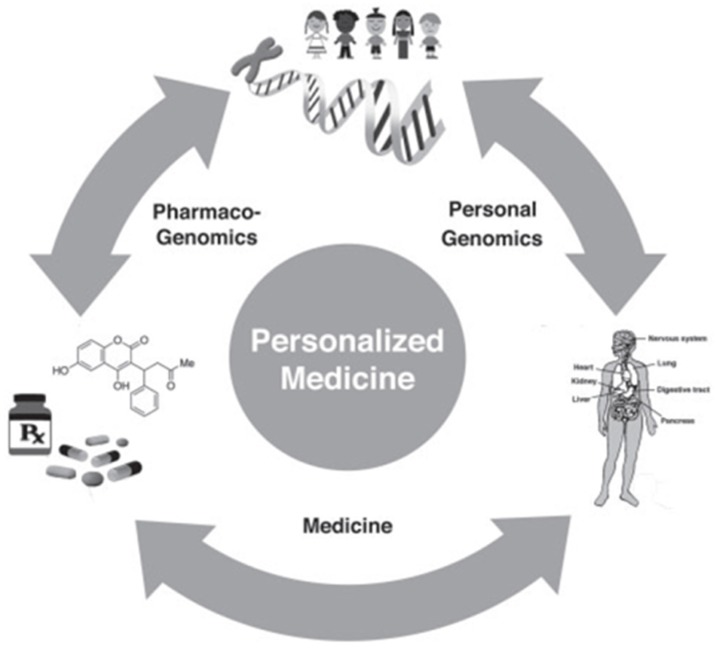
The cycle of translational genomics medicine indicating applications of personalized medicine for patient and population-based genomic studies, especially in pharmacogenomics.

The genomic research discoveries, like pharmacogenomics, *etc.*, are probably going to create a deep impact on future medical therapeutics and diagnostics [53] (Figure 3). To put it into perspective, the human genome is known to contain about 20 thousand genes [65], but it is been suggested that the current level of medications target approximately 500 genes, *i.e.*, only 2.3% of the whole genome [6] and even less is being targeted in pathogenic genomes. Scientists are excited by the potential contribution of new fields such as precision and/or personalized medicine, as well as other emerging “-omics” branches like phenomics (study of full set of phenotypes), exposomics (study of exposure of environment on human genome and health during life time, and the spatial health care system (Geographic Information Systems) in implementing a precision-based public healthcare system. Genomic discoveries are opening up new opportunities for the pharmaceutical industry, too, by identifying novel genomic/epigenomic targets. The other genetic-based tests are also being explored to find a link between genome perturbations and their linkage with environmental, as well as biological, risk factors like nutrition, aging, chemical agents, *etc.* Genomic tools, especially NGS, is emerging as a promising platform for prediction of an individual’s response to targeted therapies and could become part of routine diagnostics in the future, also, but it has yet to be evaluated in terms feasibility, standardization protocol, computational resources, cost, capacity, reliability, and reproducibility of its results with the more commonly-used diagnostic formalin-fixed, paraffin-embedded (FFPE) materials [65,66]. The system-level annotation, especially macromolecular interaction networks, will greatly help in inducting high-throughput omics data and, hence, provide new possibilities for unrealized therapies and diagnosis through human interactomics. The test for NGS-based approaches will be to improve the standardization of procedures, as well as storage, analysis, and interpretation of data, besides other ethical aspects, which will be debated in coming years [29]. One of the most daunting difficulties faced by NGS tools is the inability of physicians and patients in understanding the ways of using genomic information for healthcare benefits. The establishment of a central repository of data collection for all genome sequencing projects for data-sharing [30] and its usage for diagnostic and therapeutic purposes will be a great step in implementing genomic information for modern clinical care.

The big data generated by Next Generation Sequencing platforms are also known to be highly and poorly predictive, validated, and non-validated, and more or less probabilistic [66], therefore clinicians and other medical staff must take the utmost caution when applying genome-based applications especially in medical diagnosis and prognosis. Clinicians and other medical staff should also abreast themselves with the latest developments, as well as risk factors associated with misinterpretation of data [12]. Traditional medical geneticists are undoubtedly going to play a vital role in advancing genome-based approaches and, therefore, there is a persuasive need for various medical fraternities to embrace the latest cutting-edge genomic knowledge. Critical to the implementation of public precision medicine approach will be to educate doctors, medical professional, nurses, and other associated medical staff about the uses and benefits of genomic tools for disease prevention and therapeutics for healthy societies (Figure 2).

Initiation of a number of genomic projects like H3Africa Initiative (Human Hereditary and Health in Africa), Qatar Genome Project, Mexico National Institute of Genomic Medicine being started in low and middle-income countries is hopefully going to bring a paradigm shift in the healthcare approach in developing countries [40,66]. Therefore, implementation of large population-based genome programs in other developing countries should also be encouraged by sharing technical know-how and generous funding by different international granting agencies. The expansion and implementation of population based genomic projects in developing countries with a vast population base will be a windfall for worldwide precision public healthcare. Scientists should, thus, concentrate on how comprehensive or focused the elucidation of genome sequencing outcomes should be. Therefore, there is a need to fill the void between high-throughput sequencing and the capacity to manage, interpret, and analyze the omics data and implement it in clinical care. Appropriate infrastructure within research institutions and hospitals that will combine clinics for patient’s samples, experimental labs for sequencing, and bioinformatics labs for data analysis, interpretation, and processing of analyzed data for clinician usage should be helpful in developing effective personalized treatment strategies [17]. The major logistical difficulty will be the delivery of genome sequencing data to clinicians and how they can use and implement the same for treatment and patient care, as well as how family members will be able to understand the pros and cons of genomic medicine [67].

Other critical and ethical issues would be whether to disclose the prediction-based genomic information to parents especially for conditions that do not have immediate consequences for the health of the child in the immediate future, like adult-onset diseases, *etc*. [68]. Nevertheless, precision public healthcare initiatives will bear major upshots a few years down the line, but there should be additional remarkable feats by different omics-based methodologies sooner to further promote precision and/or personalized medicine approaches and make translational medicine a reality, not only at the individual level, but also at the general public/population levels.

## Abbreviations

NGS	Next Generation Sequencing
PGS	Personal Genome Sequencing
WG	Whole Genome
WE	Whole Exome
PGM	Personalized Genome Medicine
PGS	Personal Genome Sequencing
